# *De novo* biosynthesis of quercetin in *Yarrowia Lipolytica* through systematic metabolic engineering for enhanced yield

**DOI:** 10.1186/s40643-024-00825-w

**Published:** 2025-01-22

**Authors:** Yuxing Dong, Wenping Wei, Mengfan Li, Tao Qian, Jiayun Xu, Xiaohe Chu, Bang-Ce Ye

**Affiliations:** 1 Institute of Engineering Biology and Health, Collaborative Innovation Center of Yangtze River Delta Region Green Pharmaceuticals, College of Pharmaceutical Sciences, Zhejiang University of Technology, Hangzhou, Zhejiang 310014 China; 2 Laboratory of Biosystems and Microanalysis, State Key Laboratory of Bioreactor Engineering, East China University of Science and Technology, Shanghai, 200237 China

**Keywords:** Biosynthesis, *Yarrowia Lipolytica*, Kaempferol, Quercetin, Metabolic engineering

## Abstract

Kaempferol and quercetin possess various biological activities, making them valuable in food and medicine. However, their production via traditional methods is often inefficient. This study aims to address this gap by engineering the yeast *Yarrowia lipolytica* to achieve high yields of these flavonoids. We designed a kaempferol biosynthetic pathway by integrating multiple-copy fusion enzyme expression modules, F3H-(GGGGS)_2_-FLS, into the genome with an optimized linker (GGGGS)_2_ to enhance kaempferol production from naringenin. To synthesize quercetin de novo, we introduced the FMOCPR gene into the kaempferol-synthesizing strain using the optimized *p*FBAin promoter. Notably, increasing glucose concentration effectively boosted the production of both flavonoids. Our results demonstrated kaempferol and quercetin titers reaching 194.30 ± 7.69 and 278.92 ± 11.58 mg/L, respectively, in shake-flask cultures. These findings suggest that *Y. lipolytica* is a promising platform for the efficient production of flavonoid-derived products.

**Supplementary Information:**

The online version contains supplementary material available at 10.1186/s40643-024-00825-w.

## Introduction

Kaempferol and quercetin are naringenin derivatives with a wide range of pharmacological activities, including antioxidant, blood pressure lowering, anti-inflammatory, anticancer, neuroprotective, hepatoprotective and tissue repair properties (Tian et al. 2021; Dabeek and Marra 2019; Al-Khayri et al. 2022; Bangar et al. 2023; Jan et al. 2022; Mckay et al. 2023; Alexander et al. 2023). These compounds are extensively used in healthy foods and medicines. Traditional plant extraction is affected by seasonality, long plant growth cycle, requires a large amount of land (Borrás-Linares et al. 2015). Chemical synthesis is accompanied by the use of a large number of organic reagents, which often has problems such as complex reaction process, low yield, high cost, and environmental pollution. The Microbial synthesis has mild conditions, short synthesis cycle, cheap raw materials, low production costs, and can reduce the use of organic reagents. It offers a promising alternative, being both environmentally sustainable and season-independent.

To date, the kaempferol and quercetin biosynthesis from microbial has been achieved in various organisms, including *Saccharomyces cerevisiae* (Rodriguez et al. 2017; Trantas et al. 2009; Tartik et al. 2023), *Streptomyces* (Marin et al. 2018) and *Escherichia coli* (Qiu et al. 2022; Stahlhut et al. 2015). Previous research has focused on synthesizing these compounds from substrates like glucose (Rodriguez et al. 2017) and coumaric acid (Duan et al. 2017), employing strategies such as introducing synthetic pathways (Trantas et al. 2009), optimizing key synthetic genes (Qiu et al. 2022; Pei et al. 2019), and enhancing precursor pathways (Rodriguez et al. 2017; Trantas et al. 2009). Despite these efforts, the titers of kaempferol and quercetin remain low, the higher yields of kaempferol and quercetin reported above were 26.57 ± 2.66 mg/L, and 20.38 ± 2.57 mg/L, respectively. Among them, the titers of kaempferol and quercetin in fed-batch fermentation in *S. cerevisiae* (Tartik et al. 2023) reached a new level of 956 mg/L 930 mg/L from the phenylpropanoid acids, respectively.

Synthetic biology offers a cutting-edge approach to natural product synthesis, enabling the *de novo* production of compounds such as terpenoids, alkaloids, and flavonoids in microbial chassis (Rodriguez et al. 2017; Cravens et al. 2019; Patra et al. 2021; Liu et al. 2021). *Y. lipolytica* is particularly advantageous due to its ability to supply acetyl-CoA and malonyl-CoA and other abundant precursors(Worland et al. 2020; Miller and Alper 2019; Spagnuolo et al. 2018). High titers of malonyl-CoA-derived compounds such as naringenin (Palmer et al. 2020; Wei et al. 2020), resveratrol (He et al. 2020; Liu et al. 2022), polydatin (Shang et al. 2023), and triacetic acid lactone (Markham et al. 2018) have been synthesized in *Y. lipolytica*. Our previous study established a naringenin synthesis pathway in *Y. lipolytica* achieving the highest naringenin titers in glucose and xylose cultures (Wei et al. 2020). This provides an excellent synthetic chassis and precursor pathway for producing naringenin-derived natural products.

**Fig. 1:**
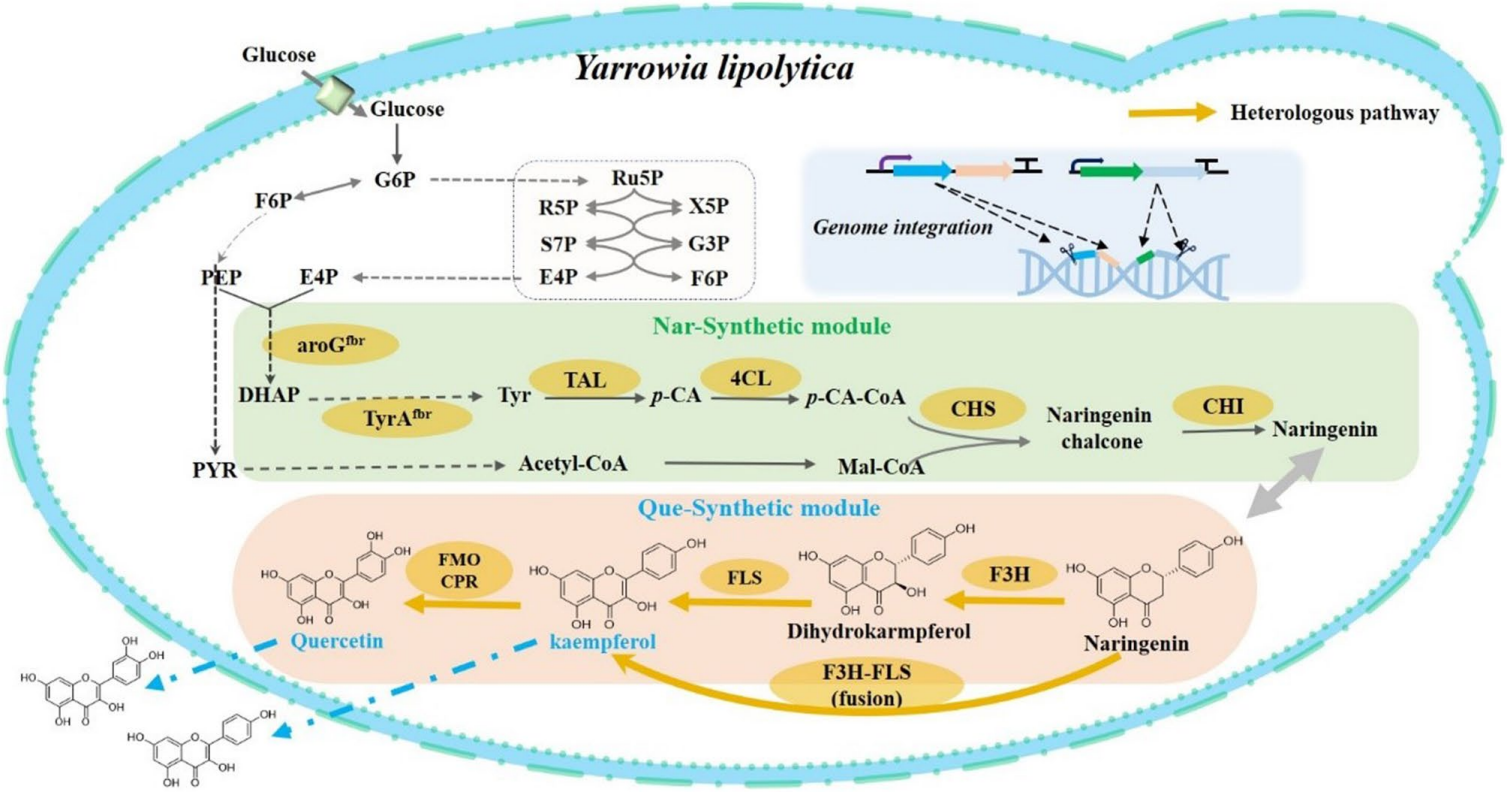
Biosynthetic pathways of kaempferol and quercetin in *Y. lipolytica*. G6P, glucose-6-phosphate; F6P, fructose-6-phosphate; PEP, phosphoenolpyruvate; E4P, erythrose-4-phosphate; DHAP, dihydroxyacetone phosphate; Tyr, L-tyrosine; *p*-CA, *p*-Coumaric acid; *p*-CA-CoA, *p*-Coumaryl-Coenzyme A; Mal-CoA, malonyl-CoA, Ru5P, Ribulose 5-phosphate; R5P, ribose-5-phosphate; X5P, D-Xylulose 5-phosphate; S7P, Sedoheptulose7-phosphate; G3P, Glycerol-3-phosphate; TAL, tyrosine ammonia lyase; 4CL, 4-coumarate: CoA ligase; CHS, chalcone synthase; CHI, chalcone isomerase; F3H, flavanone-3-hydroxylase; FMO, flavonoid 3’-monooxygenase; CPR and cytochrome P450 reductase; FLS, flavanol synthase

In this study, we aim to address the limitation of low flavonoid titers by engineering in *Y. lipolytica* for enhanced production of kaempferol and quercetin (Fig. 1). We optimized the fusion of *F3H*-*FLS* gene and integrated a genomic multi-target knock-in strategy. Additionally, we identified the most suitable promoter for expressing *FMOCPR*, enabling the *de novo* synthesis of quercetin. By increasing the initial glucose concentration in shake flask cultures, we achieved titers of 194.3 ± 8.0 mg/L for kaempferol and 278.82 ± 8.78 mg/L for quercetin. This study provides a robust approach for pathway design of kaempferol, quercetin, and other flavonoids.

## Materials and methods

### Strains, plasmids and cultivation

Plasmid construction and amplification were performed in *E. coli* DH5α. *E. coli* was cultured using Luria–Bertani (LB) liquid medium and solid medium (LB liquid medium with 20 g/L agar) containing antibiotics ampicillin (100 mg/mL) or kanamycin (50 mg/mL) 12 h at 37 °C. Engineered strains were cultured and shake flask fermented using yeast extract peptone dextrose (YPD) liquid medium (glucose 20 g/L, tryptone 20 g/L and yeast extract 10 g/L). culturation conditions, keeping the temperature at 30 °C, Rotational speed 220 rpm. Nutritionally deficient transformants were screened using yeast nitrogen base (YNB) solid medium (6.74 g/L YNB, 10 g/L glucose, and 20 g/L agar). YNB-Leu solid medium (YNB solid medium with 0.47 g/L leucine) were used when the engineered yeast lacked the Leu marker, YNB-Ura solid medium (YNB solid medium with 0.47 g/L uracil) were used when the engineered yeast lacked the Leu marker, and YNB solid medium were used when the engineered yeast contained both Leu and Ura screening markers. The URA3 screening marker was recovered using the YPD-5FOA solid medium (YPD, contain 1 g/L 5-fluoroorotic acid).

All plasmids and strains used in this study are listed in Table 1.

**Table 1:** Plasmids and strains used in this study

Name	Description	Original plasmid or strain	Source
Plasmid			
pINA1312	*Y. lipolytica*-integrative plasmid, *hp4d* promoter, XPR2 terminator, URA3 selection marker, KmR	-	Lab collection
p1312-FLS	Carrying *FLS* at *Pml* I,*Kpn* I site	pINA1312	This study
p1312-KAE-1	Carrying *FBAin-F3H-XPR2t* at *Stu* I site	p1312-FLS	This study
p1312-KAE-2	Carrying *hp4d-F3H-XPR2t* at *Stu* I site	p1312-FLS	This study
p1312-KAE-3	Carrying *hp4d-F3H(TPTP)*_*2*_*FLS* at *Pml* I, *Kpn* I site	pINA1312	This study
p1312-KAE-4	Carrying *hp4d-F3H(GGS)*_*2*_*FLS* at *Pml* I, *Kpn* I site	pINA1312	This study
p1312-KAE-5	Carrying *hp4d-F3H(GGGGS)*_*2*_*FLS* at *Pml* I and *Kpn* I site	pINA1312	This study
Linearized pHRD17	Digested by *Not* I and *Sac* II	pHRD17	Lab collection
pHRD17-KAE	Carrying *hp4d-F3H(GGGGS)*_*2*_*FLS-XPR2t* at *Spe* I site	pHRD17	This study
pHREYK1	Linearized pHR with HA and sgRNA of EYK1 inserted into *Not* I and *Sac* II site	Linearized pHR	This study
pHREYK1-KAE	Carrying *hp4d-F3H(GGGGS)*_*2*_*FLS-XPR2t* at *Nru* I site	pHREYK1	This study
pYILEU2	Carrying Leu selection marker, HA and promoter of Leu at *Not* I site	Linearized pUC57	This study
pYILEU2-QUE-1	Carrying *GPD-FMOCPR-tsyn7* at *Mlu* I and *Sac* II site	pYILEU2	This study
pYILEU2-QUE-2	Carrying *FBAin-FMOCPR-tsyn7* at *Mlu* I and *Sac* II site	pYILEU2	This study
pYILEU2-QUE-3	Carrying *hp4d-FMOCPR-tsyn7* at *Mlu* I and *Sac* II site	pYILEU2	This study
pYILEU2-QUE-4	Carrying *EXP-FMOCPR-tsyn7* at *Mlu* I and *Sac* II site	pYILEU2	This study
p1312-QUE-1	Carrying *FBAin-FMOCPR-tsyn7* at *Pml* I and *Kpn* I site	pINA1312	This study
**Strains**			
Yl-nar11	(A08):: UAS1B8TEF-*TAL-hp4d-4CL*, △DGA1(YALI0D07986); △Ku70; (Axp)::*FBAin*-*CHI-EXP-CHS;* (△myh)::*hp4d-AroG*^*fbr*^)::*hp4d-TyrA*^*fbr*^	PO1f	Lab collection
Yl-kae01	Carrying plasmid p1312-KAE-1	Yl-nar11	This study
Yl-kae02	Carrying plasmid p1312-KAE-2	Yl-nar11	This study
Yl-kae03	Carrying plasmid p1312-KAE-3	Yl-nar11	This study
Yl-kae04	Carrying plasmid p1312-KAE-4	Yl-nar11	This study
Yl-kae05	Carrying plasmid p1312-KAE-5	Yl-nar11	This study
Yl-kae06	Integration *hp4d-F3H(GGGGS)*_*2*_*FLS*-*XPR2t* into D17 site	Yl-nar11	This study
Yl-kae07	Integration *hp4d-F3H(GGGGS)*_*2*_*FLS*-*XPR2t* into EYK1 site	Yl-kae06	This study
Yl-que01	Carrying plasmid pYILEU2-QUE-1	Yl-kae06	This study
Yl-que02	Carrying plasmid pYILEU2-QUE-2	Yl-kae06	This study
Yl-que03	Carrying plasmid pYILEU2-QUE-3	Yl-kae06	This study
Yl-que04	Carrying plasmid pYILEU2-QUE-4	Yl-kae07	This study
Yl-que05	Carrying plasmid pINA1312-QUE-1	Yl-que02	This study

### Plasmid construction

Synthetic enzymes encoding the genes *F3H* (AT3G51240) and *FLS* (AT5G08640) from *Arabidopsis thaliana* (Stahlhut et al. 2015), *FMO* from *Petunia hybrida* (Rodriguez et al. 2017), and *CPR* from *Catharanthus roseus* (Rodriguez et al. 2017) were codon optimized f*or Y. lipolytica*. The sequences of the related genes are provided in the Supplementary Materials (Table S1, Table S2).

For plasmid construction, fragments were amplified using high-fidelity DNA polymerase (Vazyme, Nanjing, China) and assembled by homologous recombination using the pEASY^®^-Basic Seamless Cloning and Assembly Kit (Trans Biotech, Beijing, China). The assembled plasmids were cultured overnight in a 37 °C incubator, verified by colony PCR using Taq enzyme (Tiangen, Beijing, China), and confirmed by Sanger sequencing (Sangon Biotech, Shanghai, China).

To construct a kaempferol-producing strain, the vector pINA1312 was linearized by endonucleases *Pml* I and *Kpn* I and homologously recombined with the fragment *FLS* to obtain the plasmid p1312-FLS. Subsequently, plasmid p1312-FLS was linearized using *Stu* I and homologously recombined with fragments *pFBAin-F3H-XPR2t* and *php4d-F3H-XPR2t* to obtain plasmids p1312-KAE-1 and p1312-KAE-2, respectively. The linearized vector pINA1312 was homologously recombined with *F3H(TPTP)*_*2*_*FLS*, *F3H(GGS)*_*2*_*FLS* and *F3H(GGGGS)*_*2*_*FLS* to obtain plasmids p1312-KAE-3, p1312-KAE-4, and p1312-KAE-5, respectively. Plasmid pHRD17 was linearized using the endonuclease *Spe* I and homologously recombined with the fragment *php4d-F3H(GGGGS)*_*2*_*FLS-XPR2t* to obtain plasmid pHRD17-KAE. To construct the pHREYK1 plasmid, *Not* I and *Sac* II were used to linearize pHR, and the upstream and downstream homology arms of the EYK1 target sgRNA were homologously recombined to obtain the plasmid, pHREYK1. The plasmid pHREYK1 was linearized by the endonuclease *Nru* I and homologously recombined with the fragment *php4d-F3H(GGGGS)*_*2*_*FLS-XPR2t* to obtain the plasmid pHREYK1-KAE.

To construct quercetin-producing strains, the pUC57 backbone was digested I and homologously recombined with fragments LEU-Dn-Ter and Pleu-LEU-Ter to obtain the plasmid pYILEU2(Fig. S1). Plasmid pYILEU2 was linearized by *Mlu* I and *Sac* II, and homologously recombined with *pGPD-FMOCPR-tsyn7*, *pFBAin-FMOCPR-tsyn7*, *php4d-FMOCPR-tsyn7* and *pEXP-FMOCPR-tsyn7*, to obtain the plasmids pYILUE2-QUE-1, pYILUE2-QUE-2, pYILUE2-QUE-3, and pYILUE2-QUE-4. Plasmid pINA1312 was linearized using *Pml* I and *Kpn* I and homologously recombined with fragment *pFBAin-FMOCPR-tsyn7* to obtain plasmid p1312-QUE-1.

### Yeast transformation

Plasmids derived from pINA1312 and pYILEU2 were linearized by *Not* I, while genome integration plasmids pHRD17-KAE and pHREYK1-KAE did not require linearization. Yeast transformation was performed according to the Frozen-EZ Yeast Transformation II Kit (Zymo Research, USA) instructions, and transformed yeast was spread on YNB plates containing the corresponding nutritional deficiencies and incubated at 30 °C for 48–72 h. High-yielding strains were screened from transformants and analyzed using HPLC. The shake flask fermentation conditions were based on a previous study (Wei et al. 2020).

### Product and substrate content analysis

The titer of kaempferol and quercetin was detected by HPLC (Agilent, USA) with a DIKMA C18 column (Diamonsil Plus 5 μm C18-A, 250 × 4.6 m) at 40 °C, 10 µL injection volume, and flow rate was 1.2 mL/min. The absorption wavelengths for kaempferol and quercetin were 370 and 254 nm, respectively. Before HPLC analysis, an equal volume of anhydrous ethanol was added to the sample, centrifuged at 12,000 rpm for 2 min, and filtered using a 0.22 μm organic filter membrane. Solvent A and solvent B are the same as in the previous study (Wei et al. 2020). The analysis method followed a previous study (Wei et al. 2020). Glucose concentration was quantified using an M-100 Biosensor Analyzer (Sieman, Shenzhen China). Cellular biomass was determined by measuring the absorbance at 600 nm (OD_600_) using a Microplate Reader (BioTek, USA). Samples were diluted with water to ensure results fell within the range of 0 to 1 for glucose concentration and OD_600_ measurements.

## Results and discussion

### ***De novo*** biosynthesis of kaempferol in***Y. lipolytica***

Kaempferol biosynthesis involves the conversion of naringenin into kaempferol through the action of the enzymes F3H and FLS. F3H catalyzes the formation of dihydrokaempferol from naringenin, while FLS further converts dihydrokaempferol into kaempferol. In previous studies using *S. cerevisiae* (Rodriguez et al. 2017), *F3H* from *Astragalus mongholicus* and *FLS* from *A. thaliana* produced 26.57 ± 2.66 mg/L of kaempferol through two precursor-supplying routes. Another study (Duan et al. 2017) used *F3H* from *A. thaliana* and *FLS* from *Populus deltoides*, overexpressed the acetyl-CoA biosynthetic pathway, and supplied p-coumarate as a substrate, achieving 66.29 mg/L of kaempferol via fed-batch fermentation. A further study (Tartik et al. 2023) identified *F3H* and *FLS* from *A. thaliana* as optimal, combined with overexpression of *FLS* and increased malonyl-CoA, yielding 956 mg/L of kaempferol.

**Fig. 2:**
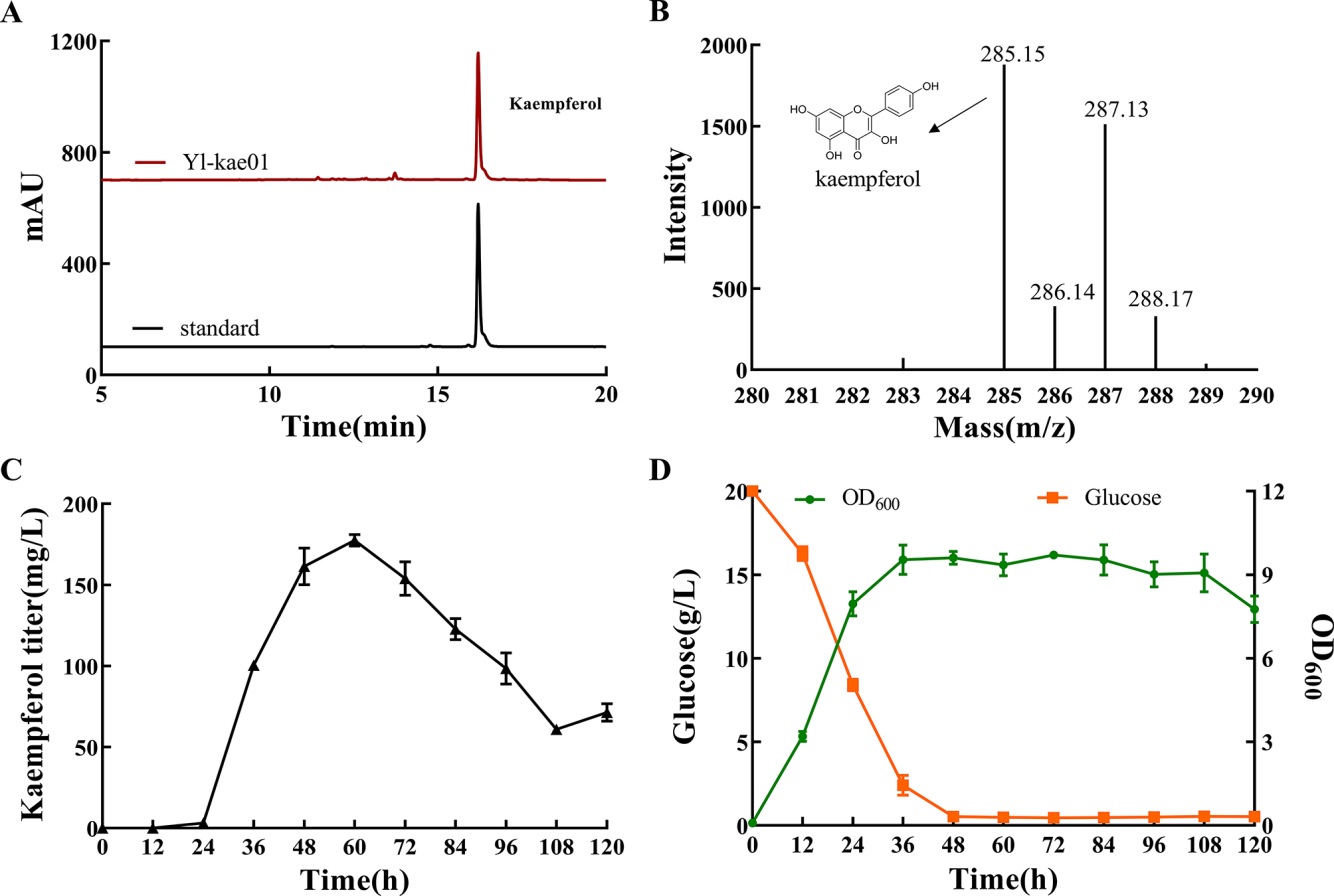
*De novo* biosynthesis of kaempferol in *Y. lipolytica*. (**A**) HPLC spectra of the fermentation broth of strain Yl-kae01. (**B**) LC-MS spectra of the fermentation broth of strain Yl-kae01. (**C**) Quantitative analysis of kaempferol titer during fermentation of strain Yl-kae01. (**D**) Time profile of glucose consumption and OD_600_ of strain Yl-kae01

In this study, a heterologous pathway for kaempferol production from glucose was established in *Y. lipolytica* by introducing the *F3H* and *FLS* genes (Fig. 1). HPLC analysis (Fig. 2A) revealed a distinct peak for kaempferol at a retention time of 16.8 min, matching the standard. The fermentation broth was extracted with ethyl acetate, and the resultant sample was analyzed by LC-MS in anion monitoring mode, showing a nucleoplasmic ratio of 285.15 (Fig. 2B), which aligns with the theoretical value for kaempferol. Structural identification by NMR also proved that the compound is kaempferol. NMR data of quercetin was presented in the Supplementary material (Fig. S2). These results confirm the successful *de novo* synthesis of kaempferol. After 60 h of fermentation, the kaempferol titer reached 177.49 ± 6.21 mg/L (Fig. 2C). Concurrently, glucose was depleted from the medium, and biomass increased significantly (Fig. 2D). The initial yield of kaempferol was higher than previously reported, likely due to the ample availability of precursors malonyl-CoA and naringenin in *Y. lipolytica*. The plasmid used for gene generation in this study incorporates a long terminal repeat zeta sequence-mediated transposon, facilitating the stable genomic integration of the *F3H* and *FLS* expression modules.

### Enhancement of synthetic capacity through fusion expression of key genes ***F3H*** and ***FLS***

**Fig. 3:**
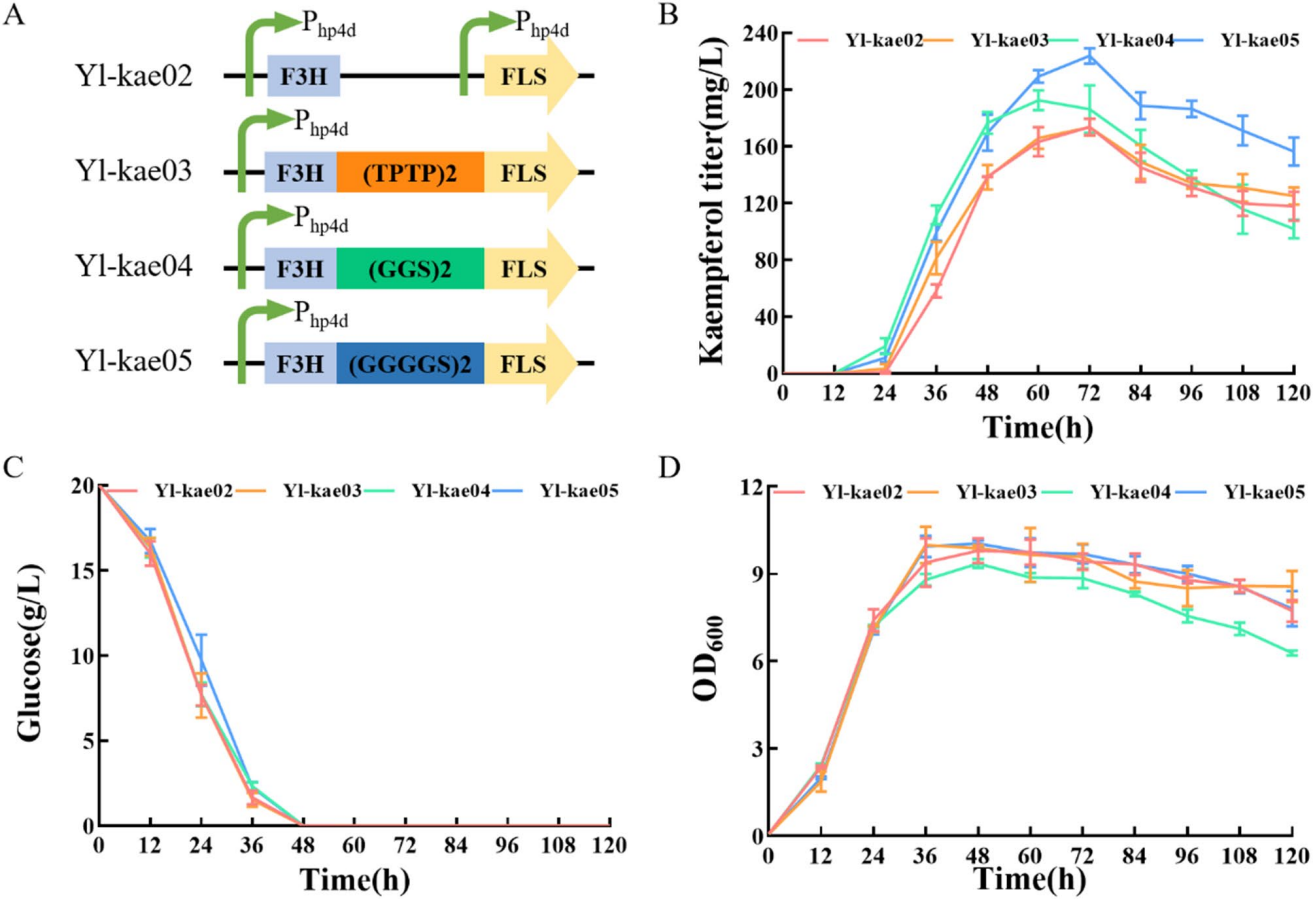
Improvement the synthetic capacity by using fusion expression of key genes *F3H* and *FLS*. (**A**) Sequences of different linkers between F3H and FLS. (**B**) Quantitative analysis of kaempferol titer during fermentation of Yl-kae02 to Yl-kae05. (**C**) Time profile of glucose consumption of Yl-kae02 to Yl-kae05. (**D**) Time profile of OD_600_ of Yl-kae02 to Yl-kae05

F3H and FLS are critical for the conversion of naringenin to kaempferol. While individually expressed enzymes can effectively catalyze this conversion, recent studies suggest that fusion enzymes can achieve higher catalytic activity (Qiu et al. 2022). This study aimed to enhance kaempferol production by constructing a fusion enzyme consisting of F3H and FLS, connected by different amino acid linkers (Fig. 3A, Table S3). The C-terminus of F3H without termination codon was fused to the N-terminus of FLS using various linkers (TPTP)_2_, (GGS)_2_, and (GGGGS)_2_ (Fig. 3A, Table S3). The catalytic performance of the fusion enzymes was compared with that of single enzyme expressions and different linker combinations. HPLC analysis of the fermentation broth after 72 h revealed that kaempferol titer increased by 1.6%, 17.98%, and 28.20% for strains using (TPTP)_2_, (GGS)_2_ and (GGGGS)_2_ linkers, respectively, compared to the control strain Yl-kae02, which expressed F3H and FLS separately under the control of hp4d. The strain with the (GGGGS)_2_ linker achieved the highest kaempferol titer of 209.21 ± 4.39 mg/L(Fig. 3B, Fig. S3).

The observed improvement in catalytic efficiency is likely due to the enhanced binding of substrates to the fusion enzyme, facilitated by the linker. Fusion enzymes can improve enzymatic activity by creating substrate channels and reducing intermediate formation. This approach effectively combines the functions and catalytic activities of both enzymes (Smith and Tsai 2007). Suitable linkers maintain the optimal distance between catalytic centers, thus enhancing overall catalytic efficiency (Chen et al. 2013). The increased efficiency may stem from the closer interaction between F3H and FLS catalytic centers. In addition, using a single promoter for the expression of both genes simplifies the construction of the expression module and enhances the efficiency of the multi-step natural product synthesis pathway.

### Genomic integration of kaempferol fusion expression modules

**Fig. 4:**
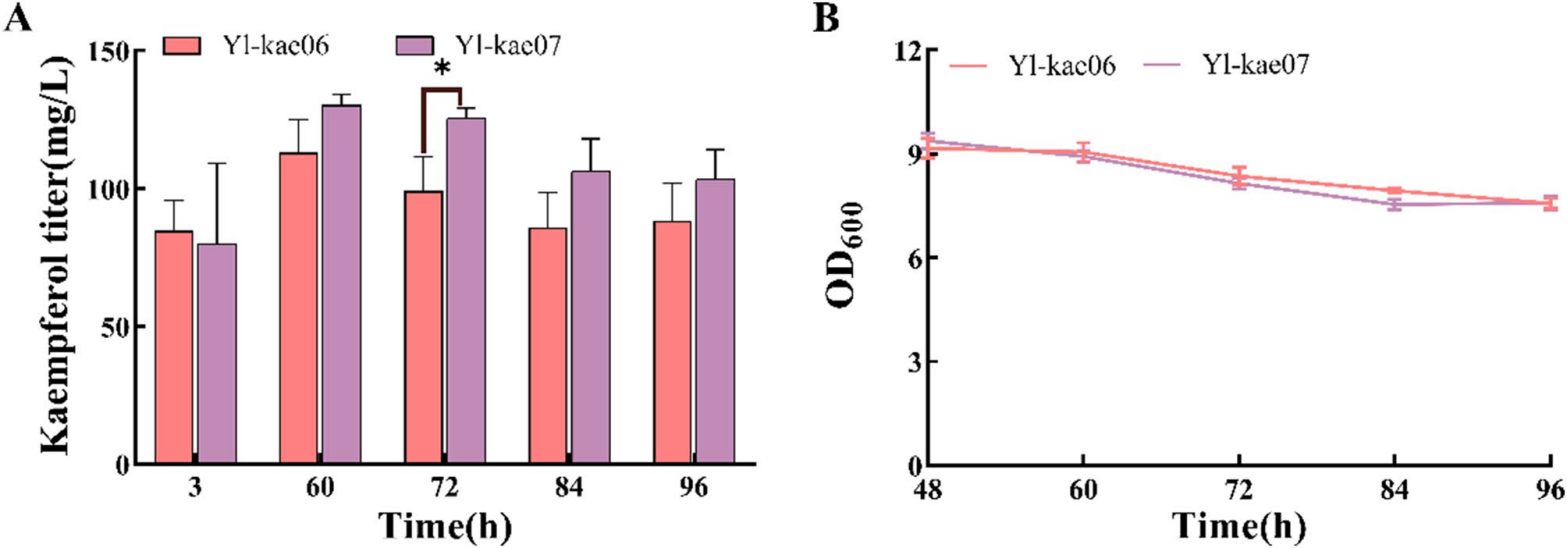
Increasing the copies of kaempferol fusion expression module in *Y. lipolytica* genome (**A**) Quantitative analysis of kaempferol titer during fermentation of Yl-Kae06 and Yl-Kae07. Data are represented as means ± SDs calculated from three independent experiments (∗*p* < 0.05; t test) (**B**) Time profile of OD_600_ of Yl-kae06 and Yl-kae07

Genomic multi-target knock-in is a widely used metabolic engineering strategy to increase product yield by inserting multiple copies of target genes at various genomic sites. In this study, we used the commonly employed *D17* locus and developed a new locus, *EYK1*(Fig. S4). Initially, the kaempferol fusion expression module was integrated into the *D17* locus to create strain Yl-kae06, which produced 113.03 ± 11.79 mg/L of kaempferol after 60 h of fermentation. Subsequently, the *EYK1* locus was established as a novel genomic insertion site (Fig. S4B). The same kaempferol fusion expression module was integrated into this locus to generate strain Yl-kae07, which yielded 130.12 ± 4.08 mg/L of kaempferol. As showed in Fig. 4A, the kaempferol titer in Yl-kae07 was 15.12% higher than that in Yl-kae06. The CRISPR-Cas9 system was employed to integrate the marker-free synthetic gene into the genome, facilitating subsequent gene knock-in and knockout operations without the need for selection markers. With strain Yl-kae07, a platform for constructing synthetic strains for kaempferol derivatives is now available. This setup allows for the future development of strains designed to produce quercetin and other related compounds.

### ***De novo*** biosynthesis of quercetin in***Y. lipolytica***

Quercetin is synthesized from kaempferol through the action of FMO and CPR. In *S. cerevisiae* (Rodriguez et al. 2017), CPR from *C. roseus* was fused with FMO from *Fragaria ananassa* and *P. hybrida* using a flexible glycine-serinelinker (5′-GGGTCGAC-3′). Among these, FMO from *P. hybrida* yielded better results. In *E. coli* (Stahlhut et al. 2015), six different sources of FMO were tested in combination with CPR from *C. roseus*. The best results were obtained using FMO from *Arabidopsis thaliana* and *P. hybrida*. Therefore, in this study, FMO from *P. hybrida* and *CPR* from *C.* roseus w*ere* selected.

**Fig. 5:**
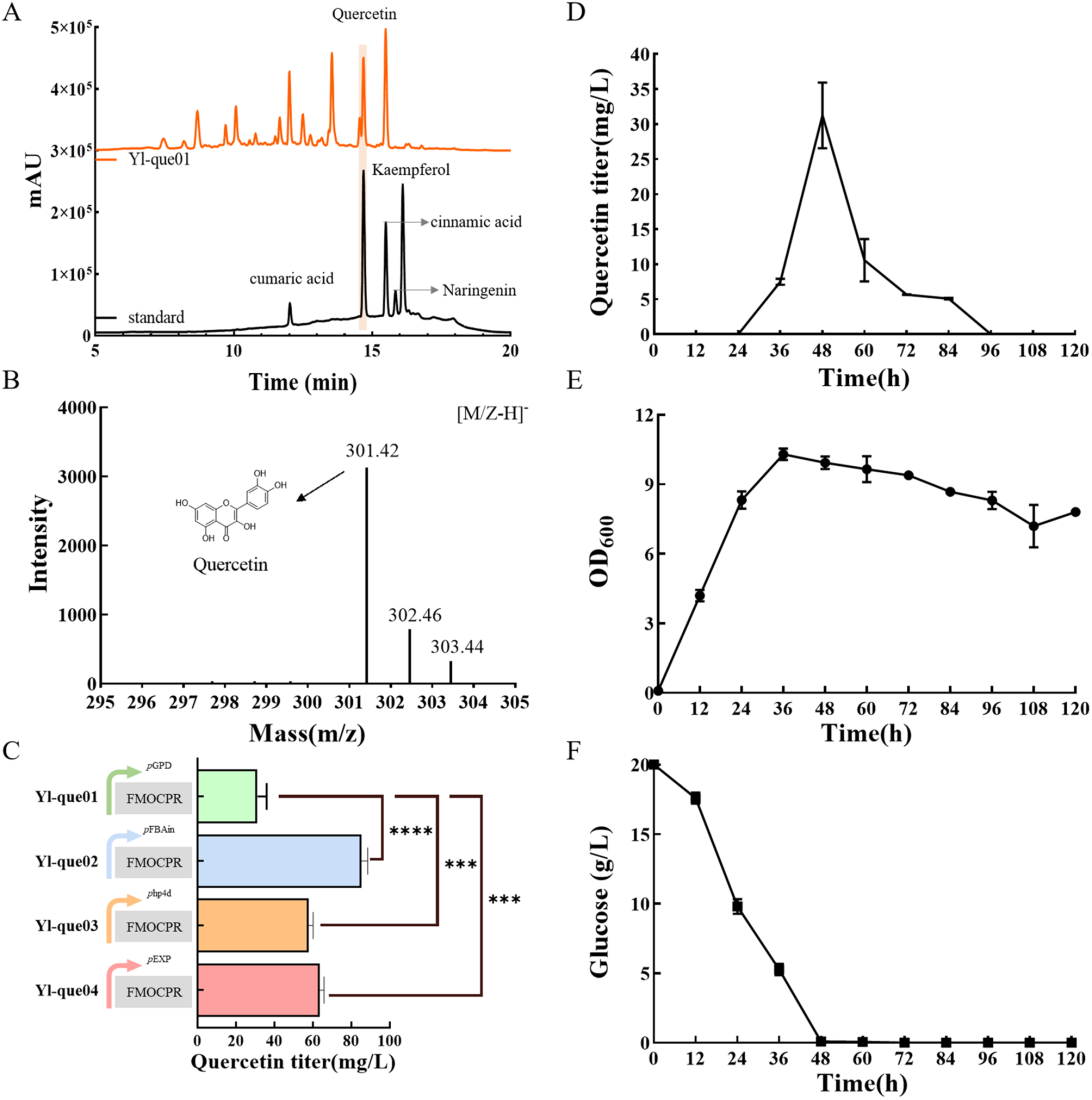
*De novo* biosynthesis of quercetin in *Y. lipolytica*. (**A**) HPLC spectra of the fermentation broth of strain Yl-que01. (**B**) LC-MS spectra of the fermentation broth of strain Yl-que01. (**C**) Comparison of quercetin titer in different promoters express *FMOCPR*. Data are represented as means ± SDs calculated from three independent experiments (∗∗∗*p* < 0.001; t test). (**D**) Quantitative analysis of quercetin titer during fermentation of strain Yl-que01. (**E**) Time profile of glucose consumption of strain Yl-que01. (**F**) Time profile of OD_600_ of Yl-que01

The quercetin synthesis module was introduced into the kaempferol-producing strain Yl-kae07 to create a quercetin-synthesizing strain. HPLC analysis revealed a specific absorption peak for quercetin at a retention time of 14.8 min, corresponding to the standard (Fig. 5A). Mass spectrometry of the purified samples in anion-monitoring mode showed a nucleoplasmic ratio of 301.42, matching the theoretical value for quercetin (Fig. 5B). Structural identification by NMR also proved that the compound is quercetin. NMR data of quercetin was presented in the Supplementary material (Fig. S5). These data confirm that the strain successfully achieved *de novo* synthesis of quercetin. After 48 h of fermentation, the quercetin titer reached 31.24 ± 4.68 mg/L. During fermentation, quercetin titer peaked at 48 h and declined rapidly thereafter. According to related reports, the reason for the titer decrease may come from the instability of quercetin itself (Stahlhut et al. 2015). Regarding the degradation of quercetin, relevant degradation genes have been identified in *Bacillus subtilis* (Niu et al. 2024). In subsequent studies, the analysis of quercetin degradation pathway or specific genes of *Y. lipolytica*, and the inactivation or knockout of these genes may be an important strategy to effectively prevent quercetin degradation and improve product accumulation.

To optimize quercetin production, different promoters (*p*EXP, *p*hp4d, and *p*FBAin) were tested for expressing *FMOCPR*, strains Yl-que02, Yl-que03, and Yl-que04 were obtained with titers of 57.82 ± 2.38 mg/L, 63.56 ± 2.29 mg/L and 85.31 ± 3.25 mg/L, respectively. Quercetin titer of strain Yl-que04 was 2.73 times higher than Yl-que01. Consequently, promoter *p*FBAin was selected for *FMOCPR* expression. Similar studies and previous research have demonstrated that intron-containing FBAin promoters are effective high-efficiency expression elements for driving the expression of key synthetic genes (Shang et al. 2023). In addition, the selected FMOCPR form demonstrated effective synthetic activity in *Y. lipolytica*, supporting the development of high-yield quercetin strains.

### Optimizing glucose concentration to improve kaempferol and quercetin titer

The synthesis of quercetin requires adequate expression of key enzymes. The absence of URA3 in strain Yl-que02 impairs its ability to synthesize uracil, which affects cell biomass accumulation and leads to suboptimal fermentation. To address this, plasmid p1312-FMOCPR, containing the FMOCPR expression module, was introduced into strain Yl-que02, creating strain Yl-que05. This modification not only enabled Yl-que05 to survive without exogenous uracil but also improved the expression of the key enzyme FMOCPR.

Additionally, we explored technological methods, including adjusting culture conditions, to enhance quercetin yield. Depletion of glucose during fermentation led to stagnation in cell growth and a corresponding plateau in product titer (Figs. 2D and 3C, and 3D). Furthermore, low product concentrations suggested possible degradation (Figs. 2C and 5D), which may result from an unknown degradation pathway or the cell using the product as a metabolic substrate when glucose is depleted. Therefore, increasing substrate supply could improve product accumulation.

**Fig. 6:**
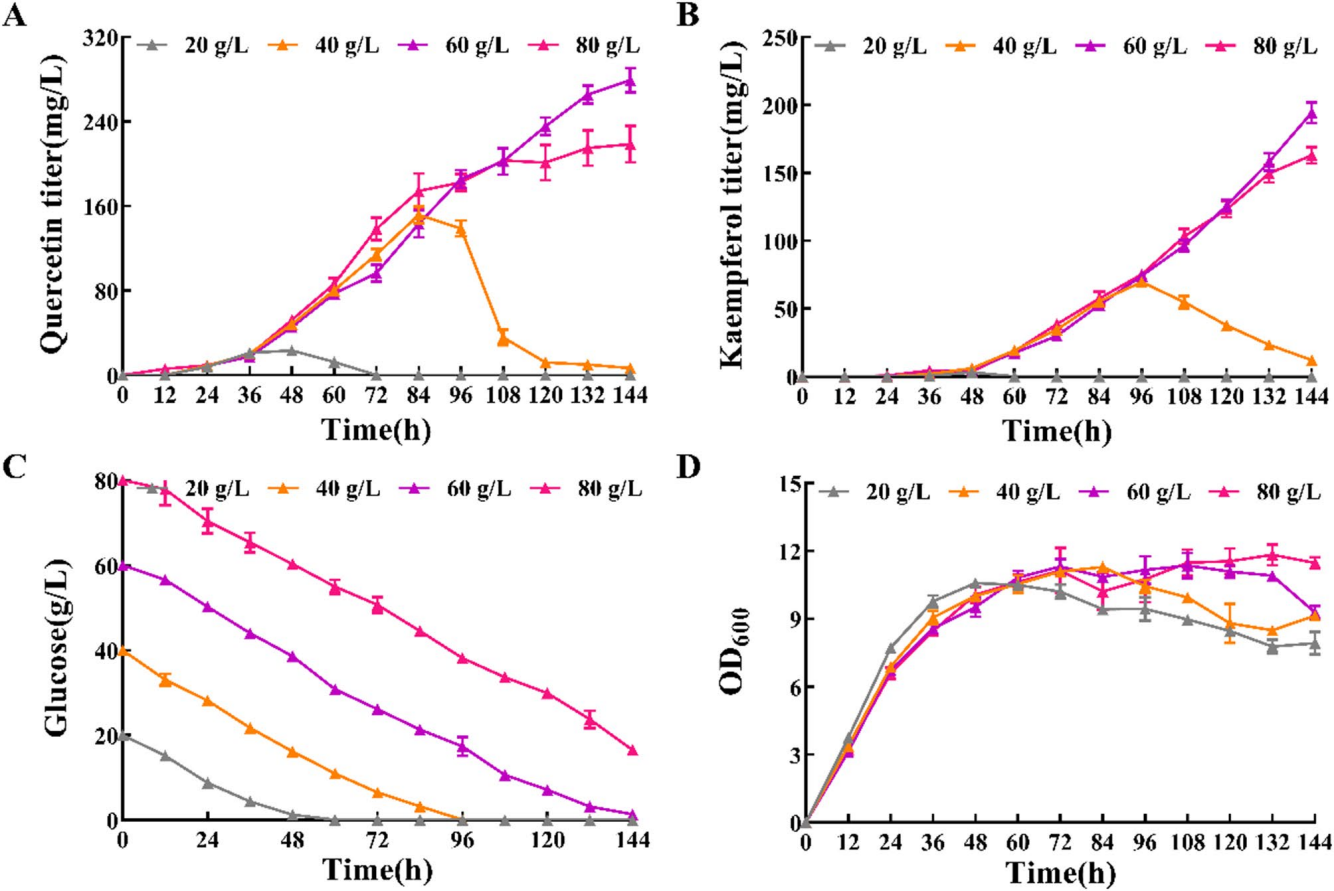
Optimizing glucose concentration to improve kaempferol and quercetin titer (**A**) Quercetin titer (mg/L) at different glucose concentrations. (**B**) Kaempferol titer (mg/L) at different glucose concentrations. (**C**) Time profile of glucose (g/L) consumption with difference glucose concentration. (**D**) Time profile of OD_600_ with difference glucose concentration

To test this, we investigated the impact of glucose concentration on quercetin production. As shown in Fig. 6A and B, strain Yl-que05 grew rapidly and produced kaempferol and quercetin during the first 48 h of fermentation. However, glucose depletion led to a cessation of kaempferol and quercetin production. Increasing glucose concentration improved production: with glucose at 80 g/L, both kaempferol and quercetin levels rose steadily. When the initial glucose concentration was increased from 20 g/L to 60 g/L, the final titers of kaempferol and quercetin reached 278.92 ± 11.58 and 194.30 ± 7.67 mg/L, respectively. Compared with relevant studies (Table S4), the de novo synthesis of glucose-derived quercetin achieved in this study has certain potential to further accelerate the development of quercetin high-yielding yeasts.

## Conclusion

In this study, we successfully constructed *de novo* biosynthesis pathways for kaempferol and quercetin in *Y. lipolytica*, achieving titers of 278.92 ± 11.58 and 194.30 ± 7.69 mg/L, respectively. Optimization of *F3H* and *FLS* linkers, genomic multi-target knock-in, promoter engineering for *FMOCPR*, and increased glucose concentration were key strategies in enhancing production. This study establishes *Y. lipolytica* as a promising host for flavonoid production and provides a foundation for further research into naringenin derivatives and other flavonoids.

## Electronic supplementary material

Below is the link to the electronic supplementary material.

Supplementary Material 1

Supplementary Material 2

## Data Availability

All data generated or analyzed during this study are included in this published article and its additional files. The authors are willing to provide any additional data and materials related to this research that may be requested for research purposes.
