# Supplementary material for: De novo biosynthesis of quercetin in Yarrowia Lipolytica through systematic metabolic engineering for enhanced yield
Source: Bioresour Bioprocess. 2025 Jan 22;12(1):5. doi: 10.1186/s40643-024-00825-w (PMC11754545; doi:10.1186/s40643-024-00825-w)
Supplement: Supplementary file 1 — Supplementary Material 1 [file 40643_2024_825_MOESM1_ESM.docx]

**Supplementary Information**

***De novo* biosynthesis of kaempferol and quercetin in *Yarrowia lipolytica* through the systemic metabolic engineering**


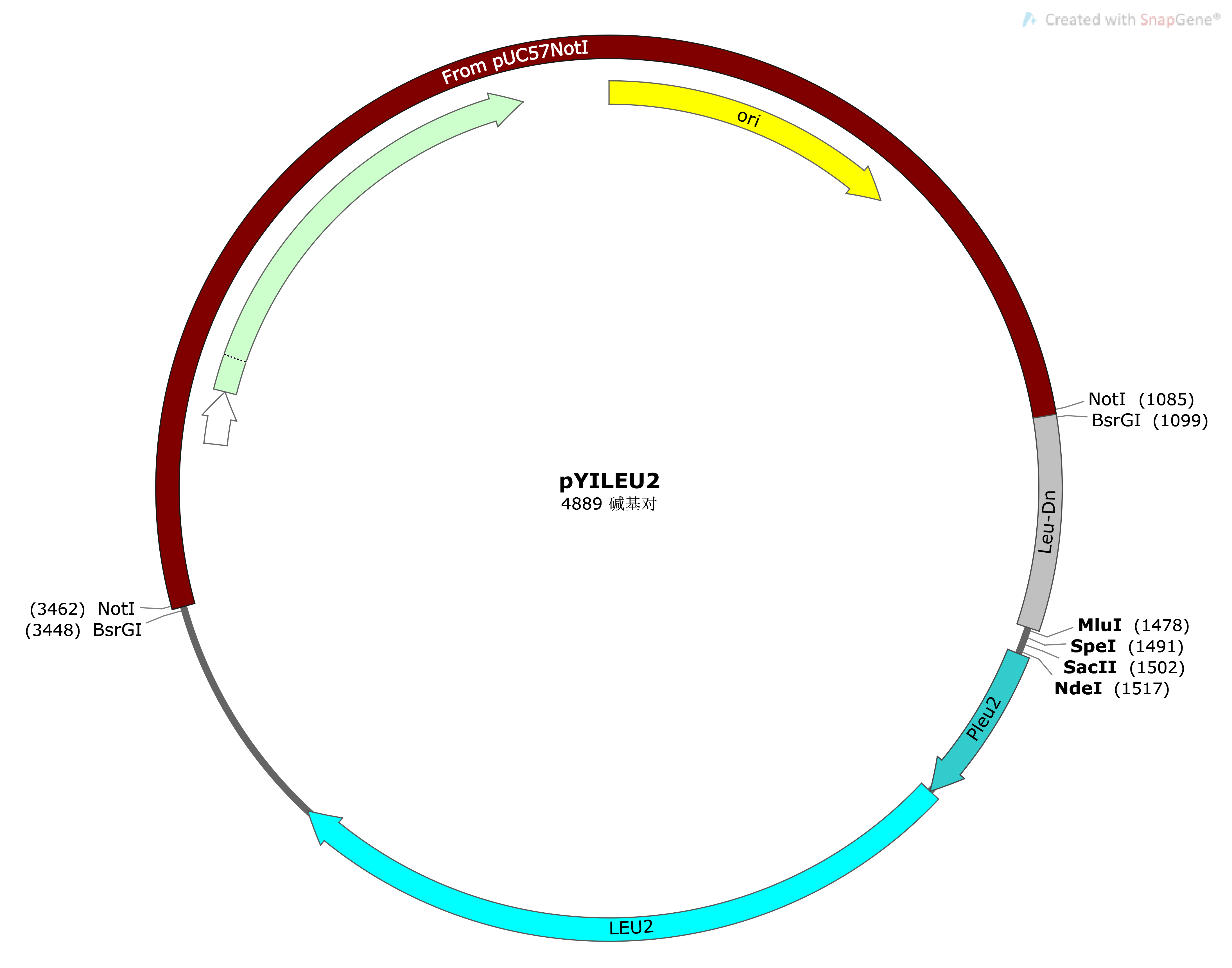


**Fig. S1**. DNA profile of plasmid pYILEU2.


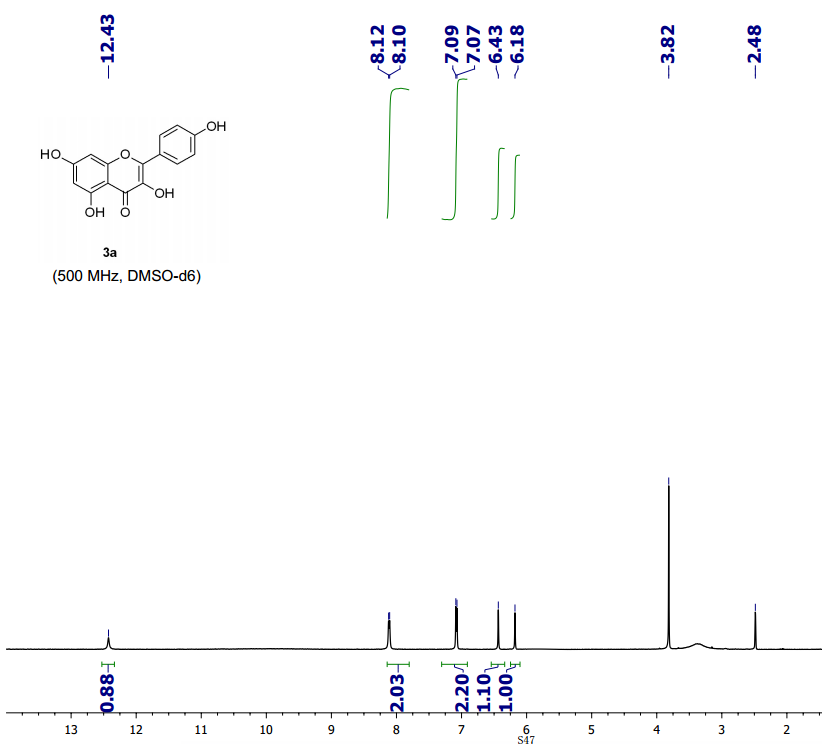


**Fig. S2.** Kaempferol 1H NMR results**.** ^1^H NMR (600 MHz, DMSO-*d*_6_) δ 12.48 (s, 1H), 10.78 (s, 1H), 10.10 (s, 1H), 9.38 (s, 1H), 8.04 (d, *J* = 8.8 Hz, 2H), 6.93 (d, *J* = 8.8 Hz, 2H), 6.44 (s, 1H), 6.19 (s, 1H).

**
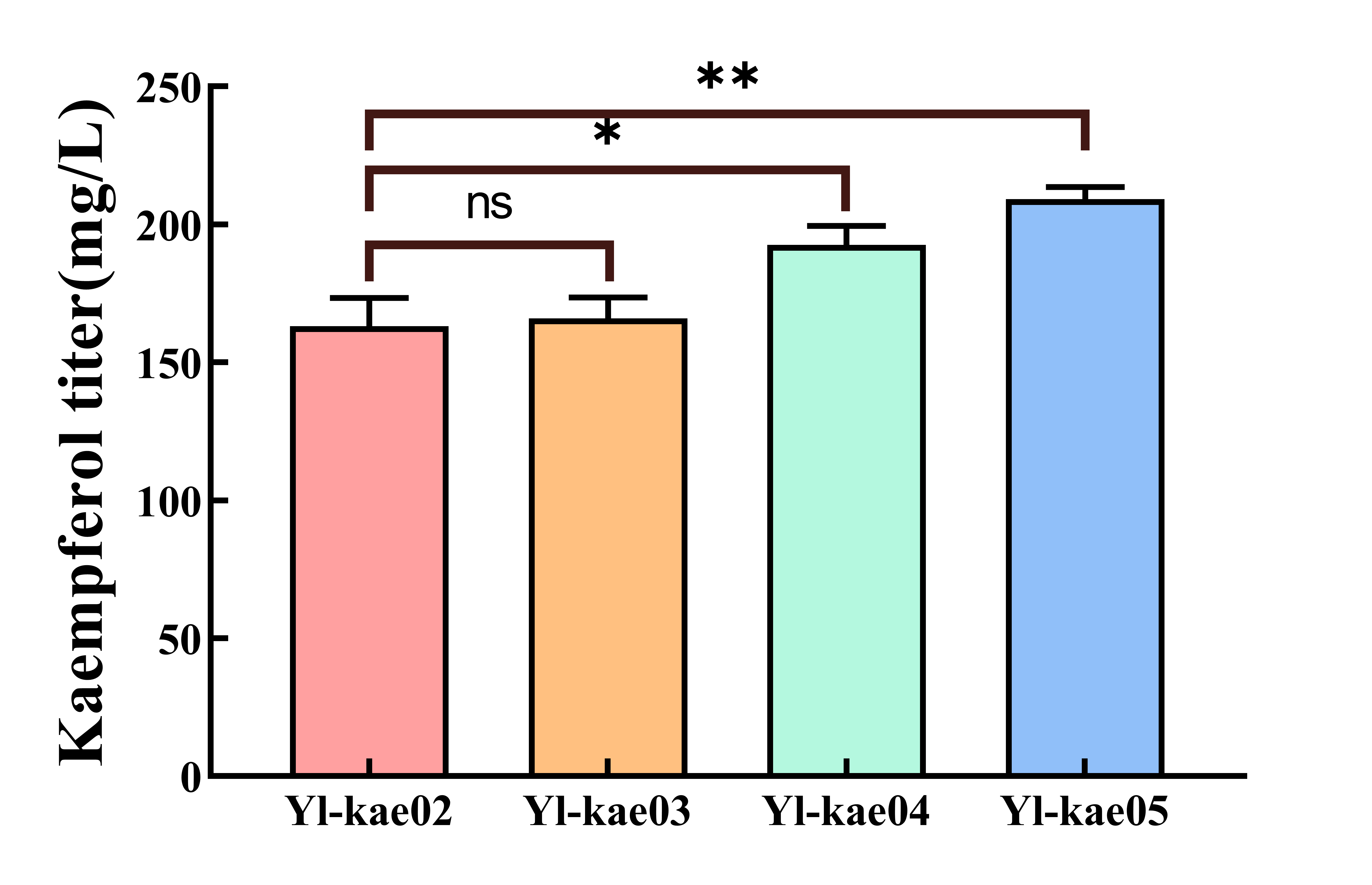
**

**Fig. S3**. Quantitative analysis of kaempferol titer of Yl-kae02 to Yl-kae05 at 72 h. Data are represented as means ± SDs calculated from three independent experiments (ns p＞0.05, ∗p < 0.05, ∗∗p < 0.01; t test)

**
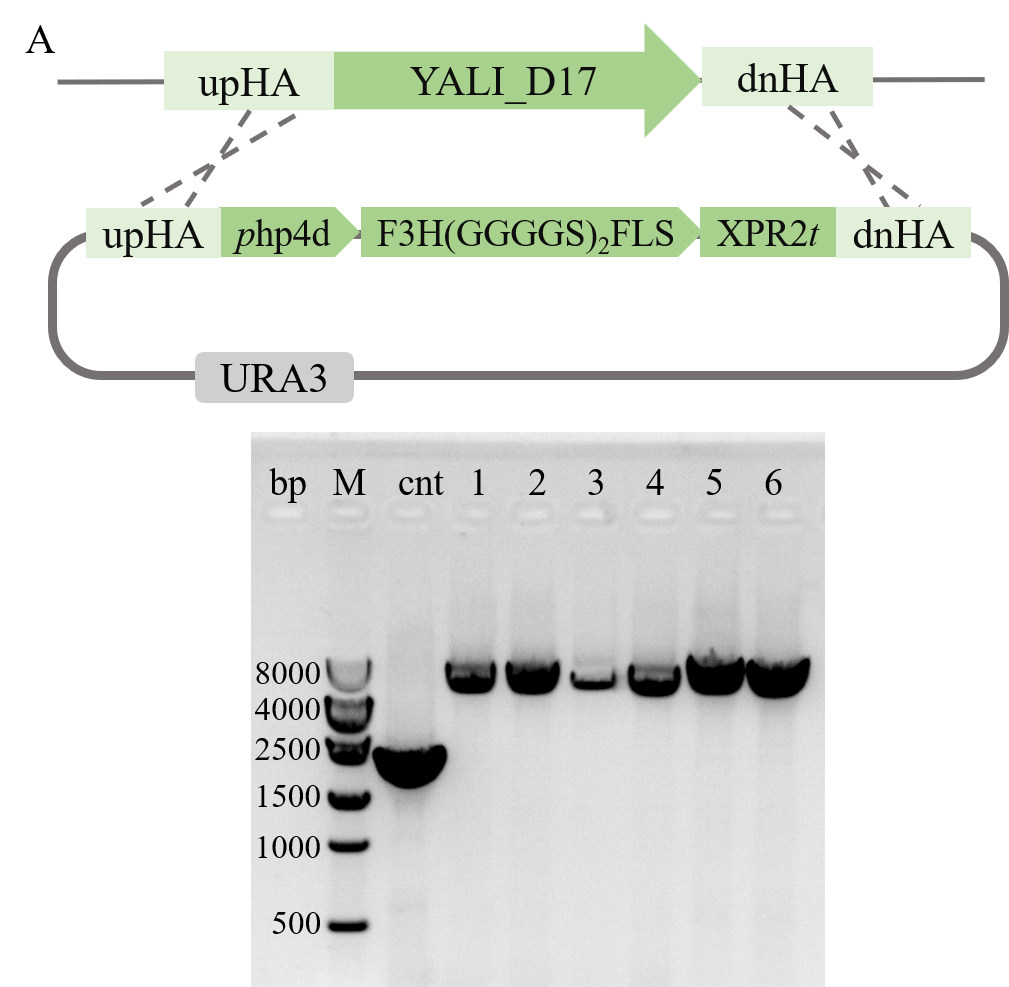
**

**
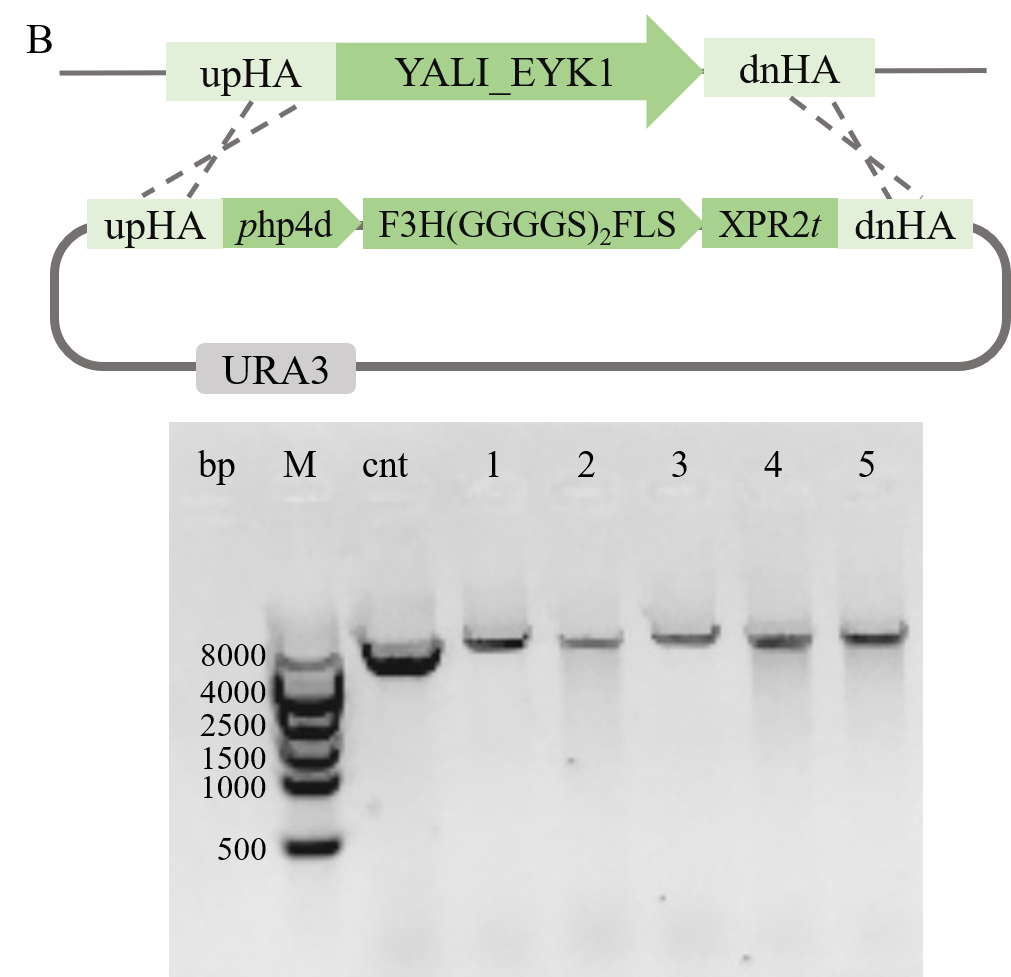
**

**Fig. S4**. Genomic PCR validation of *Y. lipolytica.* (A) Genomic PCR validation of D17 site; (B) Genomic PCR validation of EYK1 site.


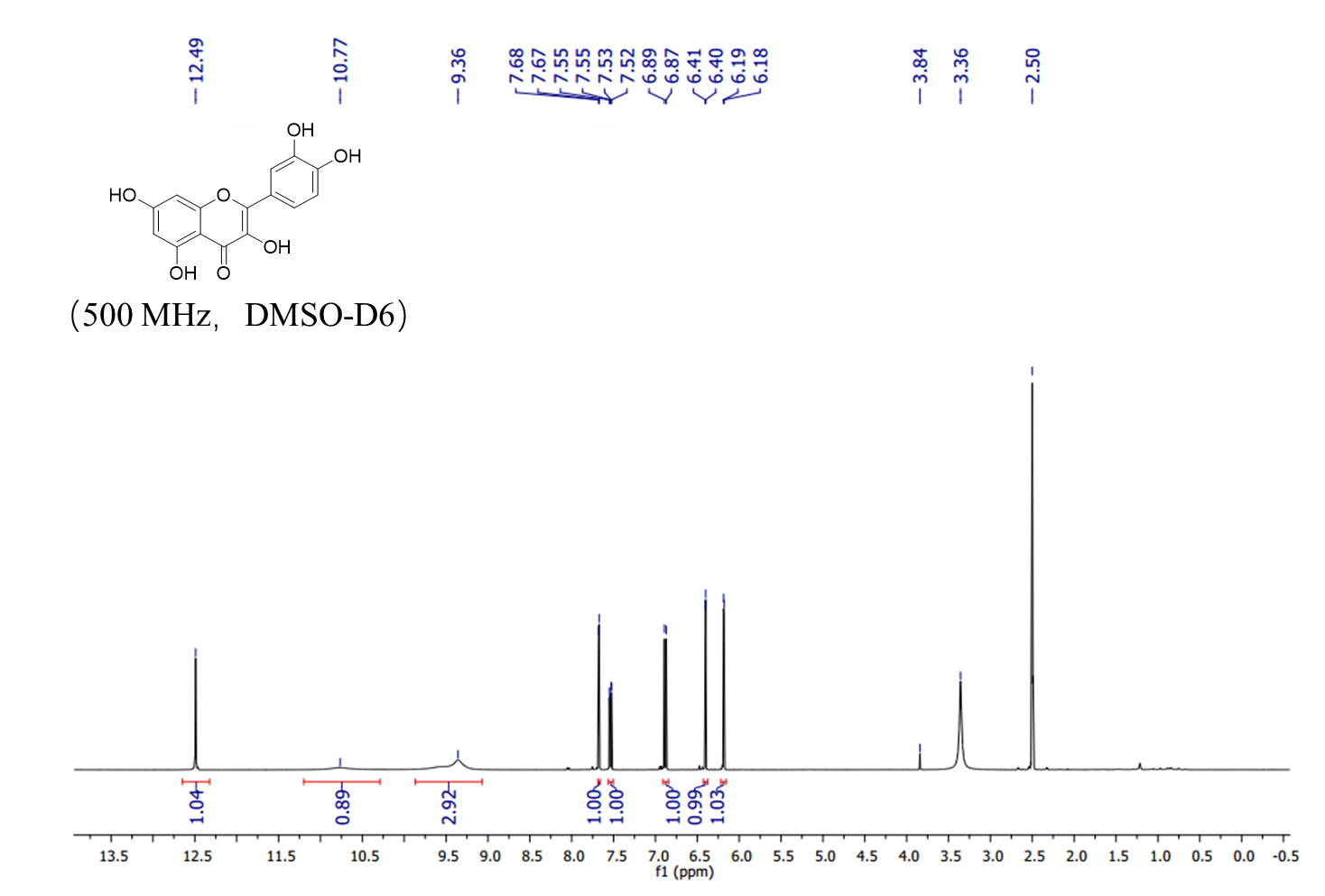


**Fig. S5.** Quercetin ^1^H NMR results ^1^H NMR (600 MHz, DMSO-*d*_6_) δ 12.49 (s, 1H), 10.78 (s, 1H), 9.58 (s, 1H), 9.34 (s, 1H), 9.29 (s, 1H), 7.67 (s, 1H), 7.54 (d, *J* = 8.5 Hz, 1H), 6.88 (d, *J* = 8.5 Hz, 1H), 6.41 (s, 1H), 6.19 (s, 1H).

**Table S1.** The primers used in this study

| Plasmids | Primers | Sequence (5′to 3′) |
| --- | --- | --- |
| p1312-FLS | FLS-0-F1 | ACATCACACATACAACCACACACATCCACGTGATGGAGGTCGAGCGAGTCCAGGA |
|  | FLS-0-R1 | ACGTGGGGACAGGCCATGGAGGTACCTTTGAAAGATGATACTCTTTATTTCTAGACAGT |
| p1312-KAE-1 | KAE-1-F1 | CACTTTGCTAGATAGAGTCGACAAAGGCCTTTTGAAAGATGATACTCTTTATTTCTAGA |
|  | KAE-1-R1 | CACACTCTCTACACAAACTAACCCAGCTCTCCATGGCCCCTGGAACCCTGACCGAGCT |
|  | KAE-1-F2 | CAGCTCGGTCAGGGTTCCAGGGGCCATGGAGAGCTGGGTTAGTTTGTGTAGAGAGTGT |
|  | KAE-1-R2 | AGCTCTGTACACCGAGAAACAGGCCTCAGTGTACGCAGTACTATAGAGGAA |
| p1312-KAE-2 | KAE-2-F1 | TAGATAGAGTCGACAAAGGGACTAGTCTGCAGCCCAAGCTAGCTTATCGATACGCGT |
|  | KAE-2-R1 | CAGGGTTCCAGGGGCCATCATCACGTGGATGTGTGTGGTTGTATGTGTGATGTG |
|  | KAE-2-F2 | ACATCACACATACAACCACACACATCCACGTGATGATGGCCCCTGGAACCCTGACCGA |
|  | KAE-2-R2 | ACGTGGGGACAGGCCATGGAGGTACCTTTGAAAGATGATACTCTTTATTTCTAGACAGT |
| p1312-KAE-3 | KAE-3-F1 | ACATCACACATACAACCACACACATCCACGTGATGATGGCCCCTGGAACCCTGACCGA |
|  | KAE-3-R1 | TGTCCTGGACTCGCTCGACCTCGGGGGTGGGGGTGGGGGTGGGGGT |
|  | KAE-3-R2 | GGGTGGGGGTGGGGGTGGCGAAAATCTGATCGACGGGCTTATCGACCT |
|  | KAE-3-F2 | ACCCCCACCCCCACCCCCGAGGTCGAGCGAGTCCAGGACATT |
|  | KAE-3-R3 | ACGTGGGGACAGGCCATGGAGGTACCTTTGAAAGATGATACTCTTTATTTCTAGACAGT |
| p1312-KAE-4 | KAE-3-F1 | ACATCACACATACAACCACACACATCCACGTGATGATGGCCCCTGGAACCCTGACCGA |
|  | KAE-4-R1 | TGGACTCGCTCGACCTCAGAACCGCCGGAACCGCCGGCGAAAATCTGATCGACGGGCT |
|  | KAE-4-F1 | ATCAGATTTTCGCCGGCGGTTCCGGCGGTTCTGAGGTCGAGCGAGTCCAGGACATT |
|  | KAE-3-R3 | ACGTGGGGACAGGCCATGGAGGTACCTTTGAAAGATGATACTCTTTATTTCTAGACAGT |
| p1312-KAE-5 | KAE-3-F1 | ACATCACACATACAACCACACACATCCACGTGATGATGGCCCCTGGAACCCTGACCGA |
|  | KAE-5-R1 | ATGTCCTGGACTCGCTCGACCTCAGAACCGCCACCGCCGGAACCGCCAC |
|  | KAE-5-R2 | ACCGCCGGAACCGCCACCGCCGGCGAAAATCTGATCGACGGGCT |
|  | KAE-5-F1 | TTCCGGCGGTGGCGGTTCTGAGGTCGAGCGAGTCCAGGACATT |
|  | KAE-3-R3 | ACGTGGGGACAGGCCATGGAGGTACCTTTGAAAGATGATACTCTTTATTTCTAGACAGT |
| pHRD17-KAE | KAE-6-F1 | TTTTCCTGTCCAAGCTAGTCTTCTATCAGGGACAGGCCATGGAGGTACCTTTGAAAGA |
|  | KAE-6-R1 | TGCTTCCGTAATATAGGTGAGACTAGTCTGCAGCCCAAGCTAGCTTATCGATACGCGT |
| pHREYK1 | EYK1-F1 | CTGTACAGACGCGTCCGCGGCAACGACTTGACTGTCTGAGCTCTAGACTTCATCA |
|  | EYK1-R1 | CCTTCGCTTCTCCTCCCTGTTAACGGTCGCGAGTGTAAGTGTGTAGAAGTGTCGTGT |
|  | EYK1-F2 | ACTTCTACACACTTACACTCGCGACCGTTAACAGGGAGGAGAAGCGAAGGAATATGA |
|  | EYK1-R2 | ATCTAGAGTTGTGTCAACTTTTGCAACTGGGCCACGGGCTGTCGTGTGTTTGACA |
|  | EYK1-F3 | AAATGTCAAACACACGACAGCCCGTGGCCCAGTTGCAAAAGTTGACACAACTCTAGAT |
|  | EYK1-R3 | TAGCTCTAAAACTGTGGCCCTGAGACTTGAGCACGTCAACCTGCGCCGACCCGGAATC |
|  | EYK1-F4 | CTCAAGTCTCAGGGCCACAGTTTTAGAGCTAGAAATAGCAAGTTAAAATAAGGCTAGT |
|  | EYK1-F4 | GCCACCTGACGTCTTTAGCGGCCGCATTCTTCGACTCTAGAGGATCTGGGCCTCGTGA |
| pHREYK1-KAE | KAE-7-F1 | ACACGACACTTCTACACACTTACACTCGCGAGGGACAGGCCATGGAGGTACCTTTGAA |
|  | KAE-7-R1 | TCCTTCGCTTCTCCTCCCTGTTAACGGCTGCAGCCCAAGCTAGCTTATCGATACGCGT |
| pYILEU2 | pLEU-F1 | ACCGAGCTCGGAATGCGGCCGCTAAAGTTTGTACAGGGTAATTGTTACAAATGATACA |
|  | pLEU-R1 | GGCCACTAGTGGATCCGACGCGTCGGACTTCATGTCACACAAACCGATCTTCGCCTCA |
|  | pLEU-R2 | GACGGAATTCCATATGGAATTCCAGATCCGCGGCCACTAGTGGATCCGACGCGTCGGA |
|  | pLEU-F2 | ACTAGTGGCCGCGGATCTGGAATTCCATATGGAATTCCGTCGTCGCCTGAGTCATCAT |
|  | pLEU-R3 | GAGTGCACCATATTTAGCGGCCGCATTCCCCTGTACAAACTAAGGTATTGAAATCCCA |
| pYILEU2-QUE-1 | QUE-1-F1 | TTTGTGTGACATGAAGTCCGACGCGTCGGGGTTGAAATGAATCGGCCGACGCTCGGTA |
|  | QUE-1-R1 | CAGAATCAGAGAGGCCATTGTTGATGTGTGTTTAATTCAAGAATGAATATAGAGAAGA |
|  | QUE-1-F2 | ATTCATTCTTGAATTAAACACACATCAACAATGGCCTCTCTGATTCTGTATACTGTGA |
|  | QUE-1-R2 | TATGGAATTCCAGATCCGCGGCCACTAGCCACTGCTTCCGTAATATAGGTGAACTAGT |
| pYILEU2-QUE-2 | QUE-2-F1 | TTGTGTGACATGAAGTCCGACGCGTCGGCAGTGTACGCAGTACTATAGAGGAACAATT |
|  | QUE-2-R1 | ACAGTATACAGAATCAGAGAGGCCATGGAGAGCTGGGTTAGTTTGTGTAGAGA |
|  | QUE-2-F2 | CTACACAAACTAACCCAGCTCTCCATGGCCTCTCTGATTCTGTATACTGTGA |
|  | QUE-1-R2 | TATGGAATTCCAGATCCGCGGCCACTAGCCACTGCTTCCGTAATATAGGTGAACTAGT |
| pYILEU2-QUE-3 | QUE-3-F1 | CGGTTTGTGTGACATGAAGTCCGACGCGTCGGCTGCAGCCCAAGCTAGCTTATCGAT |
|  | QUE-3-R1 | ATCACAGTATACAGAATCAGAGAGGCCATCACGTGGATGTGTGTGGTTGTATGTGTGA |
|  | QUE-3-F2 | TCACACATACAACCACACACATCCACGTGATGGCCTCTCTGATTCTGTATACTGTGAT |
|  | QUE-1-R2 | TATGGAATTCCAGATCCGCGGCCACTAGCCACTGCTTCCGTAATATAGGTGAACTAGT |
| pYILEU2-QUE-4 | QUE-4-F1 | TGTGTGACATGAAGTCCGACGCGTAGGAGTTTGGCGCCCGTTTTTTCGA |
|  | QUE-4-R1 | TATACAGAATCAGAGAGGCCATTGCTGTAGATATGTCTTGTGTGTAAGGGGGTT |
|  | QUE-4-F2 | ACACACAAGACATATCTACAGCAATGGCCTCTCTGATTCTGTATACTGTGA |
|  | QUE-1-R2 | TATGGAATTCCAGATCCGCGGCCACTAGCCACTGCTTCCGTAATATAGGTGAACTAGT |
| p1312-QUE-1 | QUE-5-F1 | AAGCTAGCTTATCGATACAGTGTACGCAGTACTATAGAGGAACAATT |
|  | QUE-5-R1 | ACGTGGGGACAGGCCATGGATTTGAAAAAATTTATTTCT |

**Table S2.** Gene sequence of *F3H*, *FLS* and *FMOCPR*

| **1. *F3H* sequence (5' to 3')** |
| --- |
| ctaagcgaagatttggtcgacaggcttgtcaacttctttgtggtcacgctcctctttagccagcttcttgaggcgagcaagctccaaatctcttcccatctttctcttatacatctcggcaaacgtgattggctcctccaatattgccttctctccttctcttactttcagtggatacactgtggcatccggcgcggggttctggaacgtggctatggataatctgctcgagttagagttcaccacggcctgatgatcagcattcttgaacctcccattgctcaaaaaatggccgtggtcgccgagattgacgacaaacgctccttcaacaggctgaaccgtaatccatgtcttgccattgtcacgtgtggcttgtaatccaccgacttggtcttgtagcagcaaggtaatggttccagggtcagtgtgacgcttgagtccgagggtgagatcaggctgagggcattttgggtaataattaacaacaatcttttggtccatatcgacgcatgcattggtaagagactctttctcaagacccatagcttcagacaaaacctcaagaagcttacaagccaaactcataagcctctcactatactcctccgtcactttcacccaaccttccggcttatctggccaccgtgagtagtctctgtttctcaccgggtacgagaaatacgttacaatctctctccaatcttgcacagcctctccctggaggtgactagagacgatgaatcctccttttttaccaccggacatgtcgaaacggagcttgtcttccggaggtaaagcaaagaagtcacgagcgagacgagtcatatccgccactaagttagtatcgacgccgtgatcgaccacttggaagatgccccaattctcacaagcctcaacgatctgacggcagatctctcctctttttccatcgacgtcatcgataccggcgagagagatcaccgggatttcgtcgctaaacacattgtaagcgactttgggccgttcatcttcgtccctgacaaatttagagttgagcttagactctccggctagctcagtcaaagttcctggagccat |
| **2. *FLS* sequence (5' to 3')** |
| atggttatggccggagcttcttctcttgacgagattcgacaggctcagcgagccgacggtcccgccggaattcttgctattggcaccgccaaccccgagaaccacgttcttcaggccgagtaccccgattactacttccgaatcaccaactccgagcatatgaccgacctgaaggagaagttcaagcgaatgtgcgacaagtccaccattcgaaagcgacacatgcaccttaccgaggagtttctgaaggagaaccctcacatgtgcgcttacatggctccttctctggatactcgacaggacattgtcgtcgttgaggttcctaagctgggtaaggaggccgccgttaaggctattaaggagtggggtcagcctaagtctaagatcacccacgtcgtcttttgtaccacctccggagttgatatgcccggagccgattaccagcttactaagcttctgggtcttcgaccttccgttaagcgactgatgatgtaccagcagggttgttttgccggaggcaccgtccttcgaatcgctaaggatcttgccgagaacaaccgaggagctcgagttctggttgtttgctccgagattaccgccgttacctttcgaggtccttccgatactcatctggactctctggtcggtcaggctctgttctccgacggagccgccgctcttattgttggttctgatcccgacacttccgttggagagaagcctatcttcgagatggtttccgccgctcagactattcttcccgattccgacggagctattgacggtcatcttcgagaggttggtctgactttccaccttcttaaggacgttcccggtctgatttccaagaacatcgtgaagtcccttgacgaggctttcaagcctcttggtatctccgattggaactccctcttttggattgctcatcccggaggtcccgctattcttgaccaggttgagattaagctgggtctgaaggaggagaagatgcgagctactcgacacgttctttccgagtacggtaacatgtcctccgcttgcgttctgttcatccttgacgagatgcgacgaaagtccgctaaggacggagttgctactaccggagagggtcttgagtggggagttctttttggttttggtcccggtctgaccgttgagaccgttgttcttcactccgttcctctttaa |
| **3. *FMOCPR* sequence (5' to 3')** |
| atggcctctctgattctgtatactgtgattttctccttcctgctgcagttcattctgcgatctttcttccgaaagcgataccccctgcccctgccccctggacctaagccttggcctattattggtaacctggtccacctgggtcccaagccccaccagtccaccgctgctatggcccagacctacggtcccctgatgtacctgaagatgggcttcgtcgatgtcgtcgtggccgcctctgcctccgtcgctgctcagttcctgaaaactcatgatgccaacttctcctctcgaccccccaactctggcgccgagcacatggcttacaactaccaggacctggtgttcgccccctacggccctcgatggcgaatgctgcgaaagatttgctctgtgcacctgttctctaccaaggccctggacgacttccgacacgtccgacaggacgaggtgaaaactttaactcgagccctggcctccgccggtcagaagcctgttaagctgggccagctgctgaacgtctgcaccaccaacgccctggcccgagtcatgctgggcaagcgagtgttcgccgacggttccggtgacgtcgatccccaggccgctgagttcaagtccatggtggtggagatgatggtggtggccggtgtgttcaacatcggcgacttcatcccccagctgaactggctggacattcagggcgtggccgccaagatgaagaagctgcacgcccgattcgacgccttcctgaccgacatcctggaggagcacaagggcaagattttcggtgagatgaaggacctgctgtccaccctgatttccctgaagaacgacgacgccgacaacgacggcggcaagctgaccgacaccgagattaaggccctgctgctgaacctgttcgtcgccggcaccgacacctcttcttccaccgtcgagtgggccatcgccgagctgatccgaaaccccaagatcctggcccaggcccagcaggagatcgacaaggtggtgggccgagacagactggtgggtgagctggacctggcccagctgacctacctggaggccattgtcaaggagactttccgactgcacccctctacccccctgtccctgcctcgaatcgcctccgagtcctgtgagattaacggctacttcatccccaagggttctaccctgctgctgaacgtgtgggccatcgctcgagatcctaacgcctgggccgaccccctggagttccgacctgagcgattcctgcccggcggtgagaagcccaaggtcgatgtccgaggtaacgacttcgaggtcatccccttcggtgccggtcgaagaatctgcgccggcatgaacctgggtattcgaatggtgcagctgatgatcgccaccctgattcacgccttcaactgggacctggtcagcggtcaactgcccgagatgctgaacatggaggaggcttatggtctgaccctgcagcgagccgacccccttgtggtccaccctcgacctcgactggaggcccaggcttacatcggcggctctcgacgatcttctggctccggtaagaaggtcgtggagccccccaagctgatcgtccccaagtccgtggtggagcccgaggagatcgacgagggcaagaagaagttcaccattttcttcggtacccagaccggcaccgccgagggtttcgctaaggccctggccgaggaggccaaggctcgatacgagaaggccgtgatcaaggtcattgacatcgacgactacgccgccgacgacgaggagtacgaggagaagttccgaaaggagactctggccttcttcattctggccacctacggcgacggcgagcccaccgacaacgctgctcgattctacaagtggttcgtcgagggtaacgaccgaggcgactggctgaagaacctgcagtacggcgtgttcggcctgggtaaccgacagtacgagcacttcaacaagatcgccaaggtcgtggacgagaaggtggccgagcagggtggcaagcgaatcgtccccctggtgctgggtgacgacgaccagtgcattgaggacgacttcgccgcctggcgagagaacgtctggcccgagctggacaacctgctgcgagacgaggacgacaccaccgtgtctaccacctacaccgccgccatccccgagtaccgagtcgtgttccccgacaagtccgactccctgatttccgaggccaacggtcacgccaacggctacgccaacggcaacaccgtgtacgacgcccagcacccctgtcgatccaacgtggccgtccgaaaggagctgcacacccccgcttctgaccgatcctgtacccacctggacttcgacattgccggtaccggtctgtcctacggtaccggcgaccacgtgggtgtctactgcgacaacctgtccgagactgtggaggaggccgagcgactgctgaacctcccccctgagacttacttctctctgcacgccgacaaggaggacggtacccccctggctggttcttccctgccccctcctttccccccctgcactctgcgaaccgccctgactcgatacgccgacctgctgaacacccccaagaagtctgccctgctggccctggccgcctacgcttccgatcctaacgaggccgaccgactgaagtacctggcctcccccgccggaaaggacgagtacgctcagtctctggtggccaaccagcgatctctgctggaggtgatggccgagttcccctctgccaagcccccccttggtgtctttttcgccgccattgccccccgactgcagcctcgattctactccatttcttcttccccccgaatggccccctcccgaatccatgtgacctgcgccctggtgtacgagaaaactcctggtggtcgaatccataagggcgtgtgttctacctggatgaagaacgccattcccctggaggagtctcgagactgttcctgggcccccatcttcgtgcgacagtctaacttcaagctgcccgccgaccccaaggtccccgttattatgattggccccggtaccggcctggcccctttccgaggtttcctgcaggagcgactggccctgaaggaggagggcgctgagctgggtaccgccgttttcttcttcggctgtcgaaaccgaaagatggactacatctacgaggacgagctgaaccacttcctggagattggcgccctgtctgagctgctggtggccttctcccgagagggtcccaccaagcagtacgtgcagcacaagatggccgagaaggccagcgacatctggcgaatgatctctgacggcgcctacgtctacgtctgtggcgacgccaagggcatggcccgagacgttcaccgaaccctgcacaccattgcccaggagcagggctccatggactctacccaggccgagggttttgtgaagaacctgcagatgaccggtcgatacctgcgagacgtctggtaa |

**Table S3.** Sequence of (GGS)_2_, (TPTP)_2_ and (GGGGS)_2_.

| **1. (GGS)_2_ sequence (5' to 3')** |
| --- |
| GGCGGTTCCGGCGGTTCT |
| **2. (TPTP)_2_ sequence (5' to 3')** |
| ACCCCCACCCCCACCCCCACCCCC |
| **3. (GGGGS)_2_ sequence (5' to 3')** |
| GGCGGTGGCGGTTCCGGCGGTGGCGGTTCT |

**Table S4.** Summary of the titer of kaempferol and quercetin.

| Chassis cell | Substrates | Kaempferol | Quercetin | Reference |
| --- | --- | --- | --- | --- |
| *E. coli* | Phenylpropanoid acids | 900±70 μg/L | 1100±10 μg/L | [1] |
| *S. cerevisiae* | Glucose | 26.57±2.66 mg/L | 20.38±2.57 mg/L | [2] |
| *S. cerevisiae* | Naringenin | 0.9-4.6 mg/L | 0.26-0.38 mg/L | [3] |
| *S. albus* | Glucose | Below 0.1 μM | 0.10 μM | [4] |
| *S. cerevisiae* | Glucose | 956 mg/L | 930 mg/L | [5] |

**References**

[1] Leonard E, Yan Y, koffas M A G (2006). Functional expression of a P450 flavonoid hydroxylase for the biosynthesis of plant-specific hydroxylated flavonols in *Escherichia coli*[J]. Metab Eng 8(2): 172-181.

[2] Rodriguez A, Strucko T, Stahlhut S G (2017) Metabolic engineering of yeast for fermentative production of flavonoids. Bioresour Technol 245: 1645-1654.

[3] Trantas E, Panopoulos N, Ververidis F (2009) Metabolic engineering of the complete pathway leading to heterologous biosynthesis of various flavonoids and stilbenoids in *Saccharomyces cerevisiae*. Metab Eng 11(6): 355-366.

[4] Marin L, Gutierrez-Del-Rio I, Entrialgo-Cadierno R (2018). *De novo* biosynthesis of myricetin, kaempferol and quercetin in *Streptomyces albus* and *Streptomyces coelicolor*. Plos One 13(11): e0207278.

[5] Tartik M, Liu J, Mohedano M T (2023). Optimizing yeast for high-level production of kaempferol and quercetin. Microb Cell Fact 22(1): 74.
