# Supplementary figures and images for: De novo biosynthesis of quercetin in Yarrowia Lipolytica through systematic metabolic engineering for enhanced yield
Source: Bioresour Bioprocess. 2025 Jan 22;12(1):5. doi: 10.1186/s40643-024-00825-w (PMC11754545; doi:10.1186/s40643-024-00825-w)

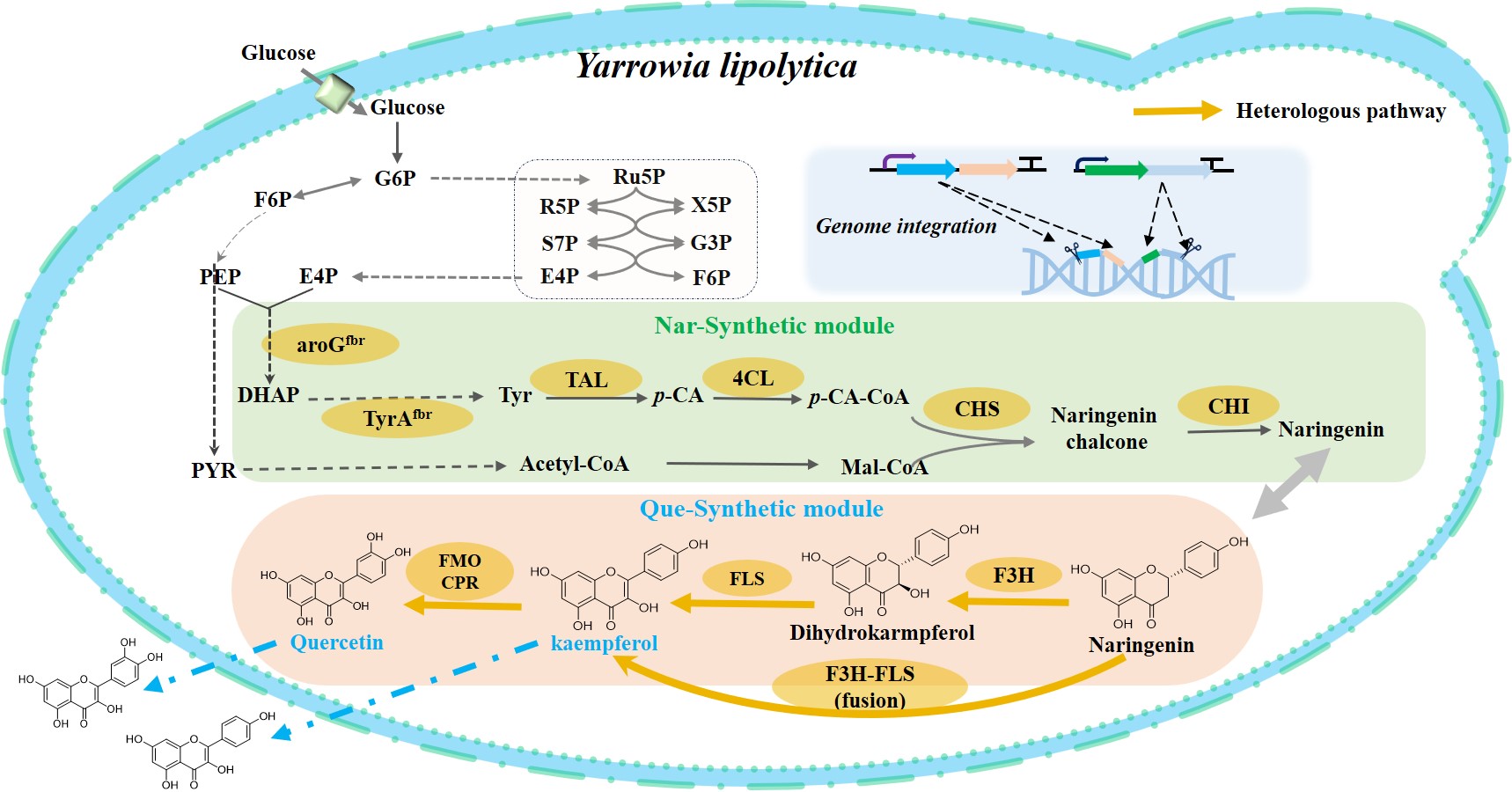

Supplement: Supplementary file 2 — Supplementary Material 2 [file 40643_2024_825_MOESM2_ESM.jpg]
